# The efficacy of ureteroscopic triage in increasing the cure rate of the first-line treatment for a ureterovaginal fistula resulting from radical hysterectomy

**DOI:** 10.1016/j.heliyon.2023.e18389

**Published:** 2023-07-20

**Authors:** Li Deng, Shuai Tang, Yuya Dou, Yudi Li, Zhiqing Liang, Yanzhou Wang

**Affiliations:** Department of Obstetrics and Gynecology, Southwest Hospital, Army Medical University (Third Military Medical University), Chongqing 400038, China

**Keywords:** Iatrogenic ureteral injuries, Ureterovaginal fistula, Ureteroscopy, Radical hysterectomy, Cure rate

## Abstract

**Study objective:**

To explore the effect of pretreatment with ureteroscopic triage for iatrogenic ureterovaginal fistula (UVF) resulting from radical hysterectomy.

**Design:**

A retrospective cohort study.

**Setting:**

Department of gynecology at a tertiary medical center.

**Patients:**

Women diagnosed with UVF secondary to radical hysterectomy at our center between April 2008 to June 2018.

**Interventions:**

The patients were divided into two groups according to whether pretreatment with ureteroscopic triage was performed. Those in the non-triage group underwent retrograde placement of a double-J stent immediately following diagnosis as the first-line therapy. Patients in the triage group were first evaluated under ureteroscopy, their ureteral injuries were then classified into different grades and then underwent different treatments as the first-line therapy, including stent placement or reconstruction surgeries. The cure rate of the first-line therapy and the timeliness of the implementation of adjuvant radiotherapy were subsequently analyzed.

**Measurements and main results:**

Ninety-eight UVF patients were included. The demographics, ECOG status, stage of cervical cancer (FIGO 2009), types and onset time of symptoms were not different between the two groups. There were 54 patients in the nontriage group, with an overall first-line cure rate of 70.4% and a timely implementation rate of adjuvant radiotherapy of 38.5%. There were 44 patients in the evaluation group, with an overall first-line cure rate of 93.2% and a timely implementation rate of adjuvant radiotherapy of 90.0%. The differences were statistically significant (p < 0.001).

**Conclusion:**

Ureteroscopic triage of ureteral injuries can guide the selection of the optimal first-line therapy for patients with UVF secondary to radical hysterectomy, increase the cure rate and ensure the timely implementation of adjuvant radiotherapy.

## 1 Introduction

An iatrogenic ureteral injury (IUI), a serious complication of gynecologic surgery, has an incidence of 0.78%, according to a large epidemiologic survey, of which 62% are not recognized intraoperatively and may result in a secondary ureterovaginal fistula (UVF) [1]. Although the incidence of this complication is rather low, its occurrence can cause considerable physical and psychological distress to the patients and is a cause of medical litigation [2]. The incidence of IUI in patients who have undergone radical hysterectomy is considerably higher than that of IUI in patients who have undergone benign gynecologic surgeries, as the operation involves dissection of the tunnel and the sheath of the pelvic course of the ureter [3]. The risk of this complication is much higher in patients with cervical cancer. Because it causes physical and psychological pain, adjuvant radiotherapy is often delayed. According to the National Comprehensive Cancer Network (NCCN) guidelines for cervical cancer, patients with moderate to high-risk factors need to undergo adjuvant radiotherapy within 6 weeks, otherwise, their survival will be negatively affected [4]. The data of patients with colon cancer were retrieved from the US national database and retrospective analysis of that data showed that a ureteral injury was independently associated with a higher mortality rate, suggesting that adjuvant postoperative treatment should be delayed [5]. Therefore, a UVF secondary to a radical hysterectomy that is treated more appropriately will increase the cure rate of the first-line treatment and reduce the impact on adjuvant therapy.

Currently, however, there is no widely accepted procedure for the treatment of iatrogenic UVF after gynecologic surgery. Some urologists recommend retrograde double J stent placement as the first-line treatment [[6], [7], [8]], however, according to the literature, the cure rate of this first-line approach is very low, and more than half of the patients undergo reconstructive surgery after prolonged observation, especially if there is thermal damage [9,10]. Such prolonged treatment is unacceptable for patients with cervical cancer who require adjuvant radiotherapy. There are also some surgeons who believe that once UVF is diagnosed, surgical reconstruction, such as a ureteroneocystostomy, can be performed immediately [11,12]. Although reconstructive surgery is a rapid curative treatment [13], patients often suffer from the trauma of re-operation, high costs, and overtreatment, especially those with mild injury. Therefore, a triage method can be used to help distinguish patients who are most suitable patients for retrograde double J stent placement from those who are most suitable for surgical reconstruction.

In 2015, we implemented the use of ureteroscopic examination for IUI diagnosis in UVF patients. Additionally, the American Association for the Surgery of Trauma (AAST) ureteral injury grading system was utilized to classify any observed ureteral damage [14]. This classification system allows us to assess and grade injuries under ureteroscopy and stratify shunt patients according to different levels of injury into different treatment processes. In this study, the cure rate of the first-line treatment for UVF secondary to radical hysterectomy before and after using ureteroscopic triage was assessed to confirm the efficacy of this strategy.

## 2 Materials and methods

### 2.1 Patients

Ninety-eight patients who were diagnosed as having UVFs at Southwest Hospital of Army Military Medical University from April 2008 to June 2018 were retrospectively included. Patients who were older than 18 years but younger than 75 years of age, with a UVF secondary to radical hysterectomy and an ECOG PS score <2 were included. The exclusion criteria were as follows: ASA III-IV; BMI>40 kg/m2; bilateral ureteral injury; and other surgical complications, such as intestinal injury, bladder injury, or postoperative bleeding. The included patients were divided into two groups according to whether they underwent ureteroscopic triage.

The study protocol was reviewed and approved by the Ethics Committee of First Affiliated Hospital of Army Medical University, and all the patients signed informed consent forms after they received counseling detailing their therapeutic options. All the procedures were performed in accordance with the relevant guidelines and regulations.

### 2.2 Treatment process

The patients were all treated immediately after UVF diagnosis. The patients in the non-triage group underwent retrograde placement of a double-J stent (COOK 4.7 Fr/26 cm) through ureteroscopy (8/12 Fr Karl-Storz) by a urologist. If placement failed, reconstruction was performed by the gynecological oncologist who performed the radical hysterectomy and the urologist.

A multidisciplinary disciplinary treatment (MDT) group composed of gynecological oncologists and urologists assessed the patients in the triage group. Under retrograde ureteroscopic examination (7.3/8 Fr Karl-Storz), ureter injuries were classified into different grades according to the AAST grading system, which was slightly modified to accommodate the characteristics of the iatrogenic injuries, as shown in Table 1. Then, patients with grade I injuries underwent retrograde double-J stent placement. Patients with grade III and above injuries underwent reconstruction surgery, mainly ureteroneocystostomy, and patients with grade II injuries underwent treatments selected by the surgeon.

**Table 1:** Description and diagram of the ureteral injury grading system of AAST based on ureteroscopy.

Grade	Description of AAST	Characteristic of IUI	Diagram
I	Hematoma (contusion or hematoma without devascularization)	Gray‒yellow necrosis mucosa and no obvious breach observed	
II	Laceration (<50% transection)	The breach and the gray‒yellow necrotic mucosa around was not more than 1/2 of the ureteral circumference.	
III	Laceration (≥50% transection)	The laceration and gray‒yellow necrotic mucosa around was more than 1/2 of the ureteral circumference	
IV	Laceration (complete transection with <2 cm of devascularization)	The ureteroscope was directly inserted into the pelvic cavity and the proximal end of the damaged ureter was not seen.	
V	Laceration (avulsion with >2 cm of devascularization)		

### 2.3 Data collection and study outcomes

Patients were identified from the hospital database, and the electronic charts were reviewed. The primary outcome was the cure rate of the first-line therapy. The patients who accepted other second-line treatments within 4 weeks were considered to have failed first-line therapy. The secondary outcome was the radiotherapy implementation rate within 6 weeks after radical hysterectomy in the patients with medium (Sedlis criteria) to high-risk factors.

### 2.4 Statistical analysis

The data were analyzed with SPSS 22.0 (IBM Corp., Armonk, NY, USA). Classified variables were reported as numbers and proportions and were analyzed by the X2 test and Fisher's exact test. Continuous variables with a normal distribution are presented as the mean ± the standard deviation (SD) and were compared using Student's *t*-test; if not, continuous variables were taken as the median and interquartile range (IQR) and were analyzed by the Mann‒Whitney U rank sum test. Differences between the groups were considered statistically significant at p < 0.05.

## 3 Results

### 3.1 Patient population

All enrolled cases were reviewed to collect information on patient demographics, ECOG status, cervical cancer stage (FIGO 2009), types and onset time of symptoms, and time interval to radiotherapy if necessary. There were 54 patients in the non-triage group and 44 in the triage group. As indicated in Table 2, there were no statistical differences in the baseline data, such as age, BMI, history of pelvic or abdominal surgeries, the stage of cervical cancer, the onset time of leakage, and so on, among the patients.

**Table 2:** The baseline clinical characteristics of the UVF patients.

Variable	Non-triage Group (n = 54)	Triage Group (n = 44)	*P*
Age (years), mean (SD)	45.2 (7.9)	46.2 (9.0)	0.592
BMI (kg/m2), mean (SD)	23.3 (3.7)	23.4 (3.4)	0.880
FIGO stage (2009), n (%)			>0.999
IA2	2 (3.7)	1 (2.3)	
IB1	16 (29.6)	14 (31.8)	
IB2	9 (16.7)	7 (15.9)	
IIA1	8 (14.8)	6 (13.6)	
IIA2	8 (14.8)	6 (13.6)	
IIB	11 (20.4)	10 (22.7)	
First symptom			0.843
Urinary leakage, n	47	38	
Fever, n	2	2	
Low intestinal obstruction, n	2	3	
Others, n	3	1	
Time to onset, mean (SD)	4 (2.3)	4 (3.7)	0.890
History of pelvic/abdominal surgery	13 (24.1)	9 (20.5)	>0.999
Need radiotherapy	25 (46.3)	21 (47.7)	0.888

### 3.2 Efficacy of the first-line therapy

**Fig. 1:**
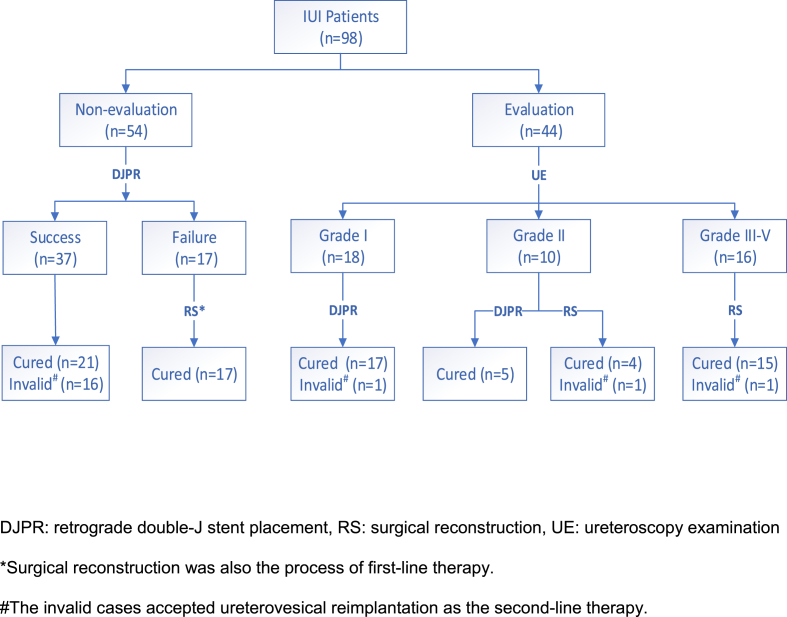
Outcomes of first-line treatment for all patients. DJPR: retrograde double-J stent placement, RS: surgical reconstruction, UE: ureteroscopy examination *Surgical reconstruction was also the process of first-line therapy. #The invalid cases accepted ureterovesical reimplantation as the second-line therapy.

As we present in Fig. 1, 37 cases (68.5%) were successfully retrograde placed with a double-J stent in the non-triage group, of which 21 were cured and 16 were invalid and later reconstructed. Seventeen cases of failed stent placement accepted a followed-by surgical reconstruction as first-line therapy. In total, the cure rate of the first-line therapy in the non-triage group was 70.3%, while the proportion of surgical reconstruction was 61.1%. Among the 25 patients needing adjuvant radiotherapy, 17 started within 6 weeks, accounting for 68%. In the triage group, there were 18, 10, and 16 patients distributed in Grades I, II, III, and above, respectively. Retrograde double-J stent placement was performed in all cases of Grade I and 5 cases of Grade II, while surgical reconstruction was accepted in 5 cases of Grade II and all cases of Grade III and above. The first-line therapy failed in 3 patients; stent placement failed in one patient and surgical reconstruction failed in 2 patients. The total cure rate of the triage group was 93.1%, which was significantly higher than that of the non-triage group. The proportion of surgical reconstruction was 50.0%, which remained basically unchanged. Among the 21 patients needing adjuvant radiotherapy, 20 started within 6 weeks, accounting for 95.2%, which was significantly higher than that in the non-triage group (Table 3).

**Table 3:** Comparison of patient outcomes with or without evaluation.

Group	Cure rate of the first-line therapy % (n/n)			Total surgical intervention rate^b^% (n/n)	Implementation rate of radiotherapy in 6w % (n/n)
	Total	retrograde double-J stent placement	surgical reconstruction		
Non-triage	70.3% (38/54)	38.3% (21/54)	100% (17/17^a^)	61.1% (33/54)	68.0% (17/25)
Triage	93.1% (41/44)	91.6% (22/24)	95% (19/20)	50.0% (22/44)	95.2% (20/21)
*P*	0.012	0.000	1.000	0.270	0.027

a The 17 cases failed retrograde double-J stent placement and accepted surgical reconstruction followed by first-line therapy.

b The proportion of cases undergoing surgical reconstruction throughout the whole therapy period.

### 3.3 Perioperative outcomes and follow-up

After 1 year of follow-up, none of the patients in the two groups had suffered from grade C-D III or above complications, including urinary leakage, ureteral obstruction, or renal inadequacy, that were secondary to the treatment of UVF. Only one patient was lost to follow-up.

## 4 Discussion

This article reports the largest study of the treatment of UVF after radical hysterectomy. The AAST grading system for ureteral injury was first introduced in the assessment of surgical complications, and the feasibility and efficacy of a ureteroscopic triage were clarified in this retrospective cohort study. The results showed that the cure rate of the first-line therapy for iatrogenic UVF secondary to radical hysterectomy improved from 70.4% to 93.2%, and the rate of radiotherapy at 6 weeks in cervical cancer patients with tumor-related risk factors increased from 38.5% to 90.0%. Moreover, the surgical reconstruction rate remained unchanged.

As mentioned before, there is no standard treatment for UVF after radical hysterectomy, so the treatment methods are generally decided by the physician. The cure rates of first-line treatments varied [10,15,16]. Overtreatment was not uncommon. Physicians often hesitate in choosing between treatment modalities, possibly choosing a treatment that is not appropriate for a specific patient. All of this is due to the lack of a sound evaluation system for triaging patients. Literature detailing methods for evaluating iatrogenic ureteral injuries is limited [17,18]. In a study evaluating the procedure of endoscopic surgery for UVF, researchers classified ureteral lesions according to their size and whether the Zebra urological guide wire could pass into the renal pelvis along the ureteral mucosa [19]. Although this method of classifying ureteral injuries was novel, it was inadequate; for example, a laceration of less than half the diameter of the ureter was not involved. On the other hand, treatments were performed under ureteroscopy only, and some of which were difficult to perform, such as a “cut-to-the-light” technique with the ureteral segments aligned via ultrasonographic and endoscopic control. Therefore, it has limited clinical application. In our study, we also used ureteroscopy as an assessment tool, but for grading, we used the AAST grading system for ureteral injury, which has been more widely recognized. Although it was originally a grading of ureteral injuries in trauma, it contains a comprehensive picture of the ureteral injuries. It is worth noting that the characteristics of iatrogenic ureteral injury vary, so modifications were made, such as replacing hematoma with gray‒yellow necrosis mucosa. Furthermore, we should assess these injuries from a development perspective. For example, for the ureter with a small breach, we need to evaluate the mucosal state around it to judge its subsequent development trend [10,20]. A gray‒yellow mucosa around indicates that delayed tissue necrosis is most likely to occur [20]. In the case of ureteral ischemia and necrosis caused by excessive dissection of the ureter, breach-like sieves can be seen under ureteroscopy, but the surrounding mucosa is usually relatively normal, predicting rapid growth and coverage. In addition, a thinner ureteroscope (7.3/8 Fr, Karl-Storz) was used to evaluate the injury to avoid secondary damage to the ureter, such as “scabbard” ureteral avulsion, ureteral wall injury, bleeding and so on [21].

According to the classification of ureteral injury of AAST, minor injuries (grade I-II) can be treated by retrograde double-J stent placement for 4–6 weeks; more severe injuries (grade III-V) usually require reconstruction [14,22]. The timing of the reconstruction has been debated for a long time. Some physicians think that the operation can be performed immediately after the onset of symptoms [23,24], while others think that it should be executed after 6–10 weeks when local inflammation or edema subsides [25,26]. Recent literature mostly supports immediate reconstruction, showing a similar success rate and shorter therapy time compared with delayed surgery [16,27,28]. Ambani et al. compared the prognosis of early repair and delayed repair in 67 patients with IUI [29]. The operation included ureteroneocystostomy and Boari flap ureteral reconstruction, and the results showed that there was no detectable difference between the two groups. The patients who needed reconstruction after evaluation in our study received an immediate operation and were 100% cured, which further confirmed the viewpoint of early reconstruction in the literature. Moreover, it is worth noting that the proportion of the patients selected for reconstruction did not increase because of the evaluation and that avoiding unnecessary treatment and observation is more beneficial for these patients. Therefore, the assessment is also a prediction of the prognosis, providing physicians and patients with more confidence in the follow-up UVF therapy and adjuvant radiotherapy.

We acknowledge the limitations of this retrospective study, including reliance on historical medical records and potential bias due to historical comparison, which may result in better outcomes in later periods due to increasing surgeon experience. Although the study is the largest of its kind worldwide, the low incidence of ureteral complications in gynecological tumor surgery results in a limited sample size. Therefore, caution is warranted when interpreting the findings, and their generalizability to the wider gynecological community requires further investigation.

In conclusion, the proper treatment for UVF secondary to the radical hysterectomy is important to cervical cancer patients, not only to improve their life quality but to also ensure timely adjuvant radiotherapy. Ureteroscopic triage, a minimally invasive procedure, is simple and highly tolerable, suggesting its high efficiency in selecting the best treatment. The AAST grading system for ureter trauma, with a little modification, can be applied to describe and evaluate iatrogenic ureteral injury more accurately. However, this report is based on empirical practice, and more cases or randomized controlled trials are needed to further prove the reliability of this assessment process.

## Ethics statement

The study protocol was reviewed and approved by the Ethics Committee of First Affiliated Hospital of Army Medical University (No. KY2020049).

## Sources of funding

None.

## Author contribution statement

Li Deng; Yanzhou Wang : Conceived and designed the experiments; Performed the experiments; Contributed reagents, materials, analysis tools or data; Wrote the paper.

Yudi Li; Zhiqing Liang: Performed the experiments.

Shuai Tang: Contributed reagents, materials, analysis tools or data.

Yuya Dou : Analyzed and interpreted the data.

## Data availability statement

Data included in article/supp. material/referenced in article.

## Declaration of competing interest

The authors declare that they have no known competing financial interests or personal relationships that could have appeared to influence the work reported in this paper.

## References

[1] Blackwell R.H., Kirshenbaum E.J., Shah A.S., Kuo P.C., Gupta G.N., Turk T.M.T. (2018). Complications of recognized and unrecognized iatrogenic ureteral injury at time of hysterectomy: a population based analysis. J. Urol..

[2] Bole R., Linder B.J., Gopalakrishna A., Kuang R., Boon A.L., Habermann E.B., Ziegelmann M.J., Gettman M.T. (2020). Malpractice litigation in iatrogenic ureteral injury: a legal database review. Urology.

[3] Barbic M., Telenta K., Noventa M., Blaganje M. (2018). Ureteral injuries during different types of hysterecomy: a 7-year series at a single university center. Eur. J. Obstet. Gynecol. Reprod. Biol..

[4] (2022). (NCCN®) NCCN. Cervical cancer. NCCN Clinical Practice Guidelines in Oncology (NCCN Guidelines®).

[5] Halabi W.J., Jafari M.D., Nguyen V.Q., Carmichael J.C., Mills S., Pigazzi A. (2014). Ureteral injuries in colorectal surgery: an analysis of trends, outcomes, and risk factors over a 10-year period in the United States. Dis. Colon Rectum.

[6] Narang V., Sinha T., Karan S.C., Sandhu A.S., Sethi G.S., Srivastava A. (2007). Ureteroscopy: savior to the gynecologist? Ureteroscopic management of post laparoscopic-assisted vaginal hysterectomy ureterovaginal fistulas. J. Minim. Invasive Gynecol..

[7] Chen Y.B., Wolff B.J., Kenton K.S., Mueller E.R. (2019). Approach to ureterovaginal fistula: examining 13 Years of experience. Female Pelvic Med. Reconstr. Surg..

[8] Rajamaheswari N., Chhikara A.B., Seethalakshmi K. (2013). Management of ureterovaginal fistulae: an audit. Int. Urogyn. J..

[9] Bahuguna G., Panwar V.K., Mittal A., Talwar H.S., Mandal A.K., Bhadoria A.S. (2022). Management strategies and outcome of ureterovaginal fistulae: a systematic review and meta-analysis. Neurourol. Urodyn..

[10] Cebeci O.Ö. (2022). Is endourological intervention a suitable treatment option in the management of iatrogenic thermal ureteral injury? A contemporary case series. BMC Urol..

[11] Tseng C.S., Tai T.E., Hong C.H., Chen C.H., Chiang I.N., Lu Y.C. (2019). Trifecta outcome of ureteral reconstruction in iatrogenic injury and non-iatrogenic ureteral lesions: a 10-year experience at a tertiary referral center. World J. Urol..

[12] Han C.M., Tan H.H., Kay N., Wang C.J., Su H., Yen C.F. (2012). Outcome of laparoscopic repair of ureteral injury: follow-up of twelve cases. J. Minim. Invasive Gynecol..

[13] Ding G., Li X., Fang D., Hao H., Li X., Zhou L. (2021). Etiology and ureteral reconstruction strategy for iatrogenic ureteral injuries: a retrospective single-center experience. Urol. Int..

[14] Coccolini F., Moore E.E., Kluger Y., Biffl W., Leppaniemi A., Matsumura Y., WSES-AAST Expert Panel (2019 Dec 2). Kidney and uro-trauma: WSES-AAST guidelines. World J. Emerg. Surg..

[15] Choi Y.S., Lee S.H., Cho H.J., Lee D.H., Kim K.S. (2018). Outcomes of ureteroscopic double-J ureteral stenting for distal ureteral injury after gynecologic surgery. Int. Urogyn. J..

[16] El Abd A.S., El-Abd S.A., El-Enen M.A., Tawfik A.M., Soliman M.G., Abo-Farha M. (2015 Dec). Immediate and late management of iatrogenic ureteric injuries: 28 years of experience. Arab. J. Urol..

[17] Ahn C.B., Kim J.H., Park G.K., Park K.Y., Bao K., Lee J.W. (2019). Prognostic imaging of iatrogenic and traumatic ureteral injury by near-infrared fluorescence. Quant. Imag. Med. Surg..

[18] Klett D.E., Mazzone A., Summers S.J. (2019). Endoscopic management of iatrogenic ureteral injury: a case report and review of the literature. J. Endourol. Case Rep..

[19] Li X., Wang P., Liu Y., Liu C. (2018). Minimally invasive surgical treatment on delayed uretero-vaginal fistula. BMC Urol..

[20] Selli C., Turri F.M., Gabellieri C., Manassero F., De Maria M., Mogorovich A. (2014 30). Delayed-onset ureteral lesions due to thermal energy: an emerging condition. Arch. Ital. Urol. Androl..

[21] Tanimoto R., Cleary R.C., Bagley D.H., Hubosky S.G. (2016). Ureteral avulsion associated with ureteroscopy: insights from the MAUDE database. J. Endourol..

[22] Bryk D.J., Zhao L.C. (2016). Guideline of guidelines: a review of urological trauma guidelines. BJU Int..

[23] Ahn M., Loughlin K.R. (2001). Psoas hitch ureteral reimplantation in adults--analysis of a modified technique and timing of repair. Urology.

[24] Ghali A.M., El Malik E.M., Ibrahim A.I., Ismail G., Rashid M. (1999). Ureteric injuries: diagnosis, management, and outcome. J. Trauma.

[25] Selzman A.A., Spirnak J.P. (1996). Iatrogenic ureteral injuries: a 20-year experience in treating 165 injuries. J. Urol..

[26] Riedmiller H., Becht E., Hertle L., Jacobi G., Hohenfellner R. (1984). Psoas-hitch ureteroneocystostomy: experience with 181 cases. Eur. Urol..

[27] Weigand K., Kawan F., Schaarschmidt T., Fornara P. (2018). Ureter complications: a rare complication but which requires the highest degree of management expertise. Urol. Int..

[28] Miyauchi K., Wada N., Nagabuchi M., Ishikawa M., Makino S., Abe N. (2022). Treatment outcome of ureteral reconstruction surgery. Hinyokika Kiyo.

[29] Ambani S.N., Skupin P., Malaeb B.S., Barboglio-Romo P., Stoffel J.T. (2020). Does early ureteroneocystostomy after iatrogenic ureteral injury jeopardize outcome?. Urology.

